# Current News

**Published:** 1893-05

**Authors:** 


### Current News.

### ILLINOIS STATE DENTAL SOCIETY AND IOWA STATE DENTAL SOCIETY—JOINT MEETING.

The Twenty-ninth Annual Meeting of the Illinois State Dental Society will be held at Rock Island, May 9 to 12, inclusive. The Thirtieth Annual Meeting of the Iowa State Dental Society will be held at Davenport, May 9 to 12, inclusive. These cities are located on opposite sides of the Mississippi River, and arrangements will be made to hold the meeting jointly, so that those in attendance at the meeting of either society will have an opportunity to listen to the papers, take part in the discussions, and witness the clinics of both societies. No efforts will be spared to make this union meeting one of the most interesting in the history of each society. Members of both societies are urgently requested to attend. All dentists are cordially invited to be present. Every one should bring models, specimens, appliances, or anything that may be of interest to the profession.

LOUIS OTTOFY,
*Secretary Illinois State Dental Society, Chicago.*
W. O. KULP,
*Chairman Executive Committee Iowa State Dental Society, Davenport, Iowa.*

---

### THE DENTAL SOCIETY OF THE STATE OF NEW YORK.

THE above Society will celebrate its twenty-fifth anniversary with a three days' session, Albany, May 10, 11, and 12. The usual number of essays and discussions by prominent men in the profession, historical reminiscences, etc., together with a dinner, will constitute the programme.

It is intended to make it rather a social than a scientific meeting, and it is hoped that a large number of the profession, both in and outside of the State, will be present. For any information regarding the meeting, address the Secretary, Charles S. Butler, Buffalo, N. Y.

---

### MASSACHUSETTS DENTAL SOCIETY.

THE Twenty-eighth Annual Meeting of the Massachusetts Dental Society will be held in Huntington Hall, Institute of Technology, Boston, on Thursday and Friday, June 8 and 9, 1893. The Society will have as its guest Professor Harrison Allen, M.D., of Philadelphia, who will give an address on this occasion ; and other prominent men are expected to participate.

Members of the profession are cordially invited to be present.

Per order of Executive Committee.

EDGAR O. KINSMAN,
*Secretary of Society.*

CAMBRIDGE, MASS.

